# Comparison of implant failure rates of different plates for midshaft clavicular fractures based on fracture classifications

**DOI:** 10.1186/s13018-019-1259-x

**Published:** 2019-07-16

**Authors:** Yung-Cheng Chiu, Kui-Chou Huang, Cheng-Min Shih, Kun-Tsan Lee, Kun-Hui Chen, Cheng-En Hsu

**Affiliations:** 1 Department of Orthopedics, China Medical University Hospital, Taichung, Taiwan; 2 School of Medicine, China Medical University, Taichung, Taiwan; 3 Department of Orthopedics, Asia University Hospital, Taichung, Taiwan; 4 Department of Occupational Therapy, Asia University, Taichung, Taiwan; 5 Department of Orthopedics, Taichung Veterans General Hospital, Taichung, Taiwan; 6 Institute of Biomedical Engineering, National Chiao Tung University, Hsinchu, Taiwan; 7 Department of Veterinary Medicine, College of Veterinary Medicine, National Chung Hsing University, Taichung, Taiwan; 8 Department of Biomedical Engineering, Hung Kuang University, Taichung, Taiwan; 9 Sports Recreation and Health Management Continuing Studies-Bachelor’s Degree Completion Program, Tunghai University, Taichung, Taiwan

**Keywords:** Dynamic compression plate, Locking plate, Midshaft clavicular fracture, Open reduction and internal fixation, Reconstruction plate

## Abstract

**Backgrounds:**

The aim of our study was to investigate failure rates of reconstruction plate and non-reconstruction plate, and find the best strategy for implant selection for different fracture types for midshaft clavicular fractures.

**Patients and methods:**

Two hundred twenty-six consecutive patients with midshaft clavicular fractures who received open reduction and plate fixation during Jan 2012 to July 2017 were reviewed. The correlations between implant failure rates and risk factors including demographic data, fracture classifications, and implant types were analyzed.

**Results:**

AO/OTA fracture classification and plate types are the most important factors affecting implant failure for midshaft clavicular fractures. Reconstruction plate had a significantly higher failure rate (53%) than that of non-reconstruction plates (3%) in comminuted midshaft clavicular (AO/OTA 15-2C) fractures (*P* value < 0.01). However, the difference was not significant in AO/OTA 15-2A and 2B classifications.

**Conclusion:**

Patients with comminuted midshaft clavicular (AO/OTA 15-2C) fractures treated with reconstruction plates had very high implant failure rates compared to non-reconstruction plates. We suggested that patients with comminuted midshaft clavicular (AO/OTA 15-2C) fractures treated with reconstruction plates need more protection and more frequent follow-up in the postoperative period.

## Introduction

Midshaft clavicular fractures were traditionally treated non-operatively, although they were associated with a lower nonunion rate compared with operative treatment [1]. However, recent multicenter, randomized control studies reported that open reduction and internal fixation (ORIF) had much lower nonunion and malunion rates as well as better shoulder function compared with non-operative treatment [2–4]. Thus, ORIF with plates is considered to be the gold standard treatment for midshaft clavicular fracture. Though ORIF effectively improves treatment outcomes, complications such as infection, hardware irritation, and implant failure diminish both patient satisfaction and functional outcome of the operation [4]. Implant failure is a major complication which often results in nonunion, malunion, and reoperation for midshaft clavicular fractures [5, 6].

Implant failure for midshaft clavicular fractures may be due to bending, breakage of plates, or screw dislodgement. Its incidence varies widely from 0 to 12.6% among the different fracture classifications and plates in the literature [5, 7–10]. However, there are few precise data on the implant failure rates of each plate based on different fracture classifications.

Four major types of plates are used for midshaft clavicular fracture fixation, as follows: reconstruction plates, locking reconstruction plates, dynamic compression plates (DCPs), and pre-contoured locking compression plates (LCPs).

Reconstruction plates, which are available in a locking and non-locking design, have a low profile and notched edges that allow easy contouring to fit the anatomical shape of the clavicle. However, the decreased plate stiffness may also reduce its resistance to fracture deformity force, which may result in yield implant failure.

DCPs are straight and stronger than reconstruction plates, but it is difficult to fit them to the anatomic S-shape of the clavicle, which may lead to implant prominence and irritation. There is potential risk of screw loosening when bone and plate are unmatched [11]. Recently, anatomically pre-contoured LCPs were introduced. They allow the application of a plate onto the clavicle without bending. LCPs induce less irritation and are more resistant to deformity force which may decrease the failure rate.

The purpose of our study was to investigate failure rates of reconstruction plate and non-reconstruction plates and find the best strategy for implant selection for different fracture types for midshaft clavicular fractures.

## Materials and methods

### Patient enrollment

The study was performed according to international standard. All authors of the present study conducted this research ethically according to international standard as required by the journal [12]. The design of this study has also been proved by our local Ethics committee (number IRBTCVGHCE17162B).

**Fig. 1:**
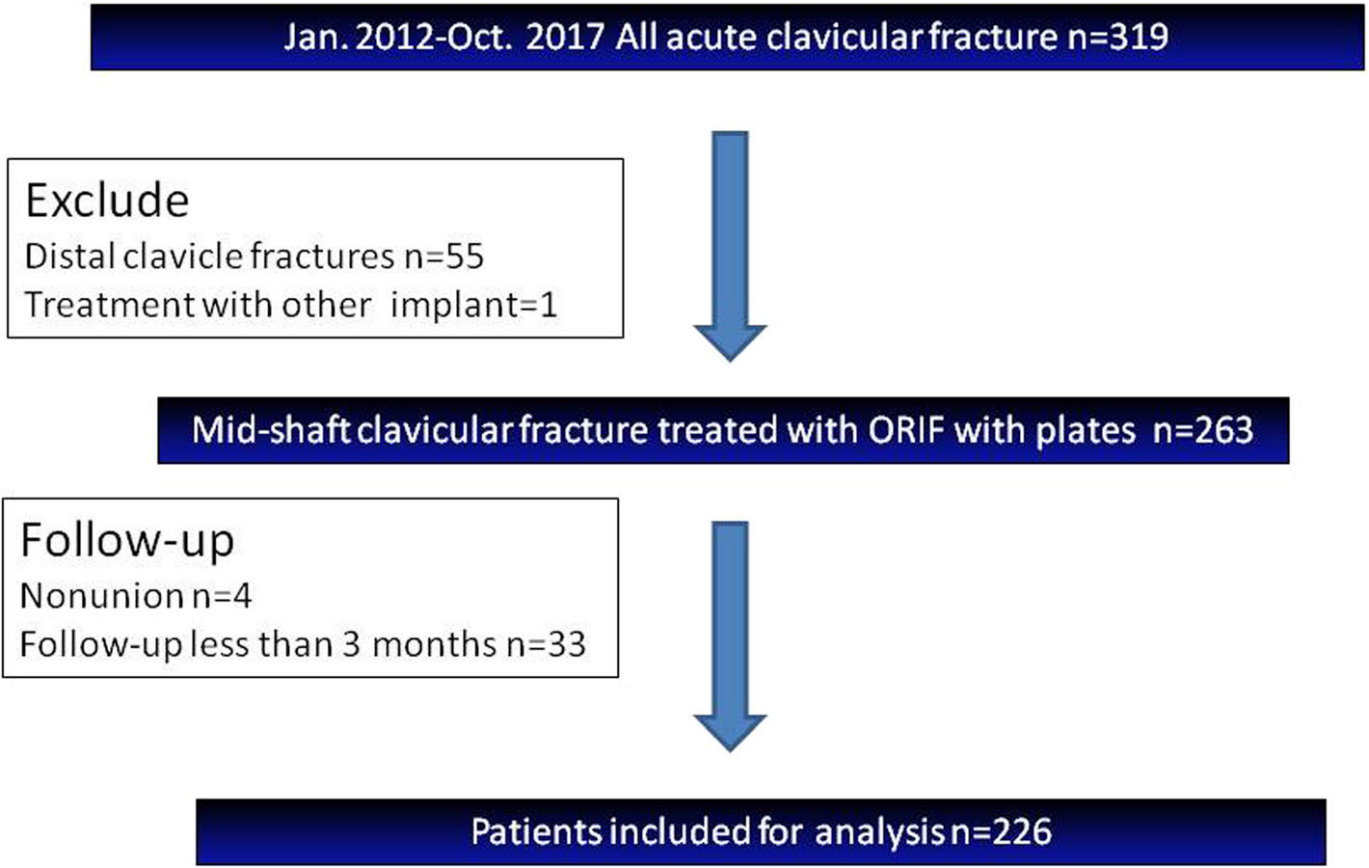
The flowchart of patient enrollment

The medical records of patients with a clavicle fracture who were treated with open reduction and internal fixation in our department between January 2012 and July 2017 were reviewed retrospectively. Patients were identified using the procedure code for open reduction and internal fixation of clavicular fractures. The indications for ORIF were mid-third clavicle fracture with more than one shaft width of displacement, more than 2 cm shortening, and impending open fractures. The exclusion criteria were pathologic fracture, previous fracture at the same region, fracture nonunion, open fractures, and the follow-up time was shorter than 12 weeks. All clavicular shaft fractures were classified according to the AO/OTA classification system. The chart of patient enrollment is shown in Fig. 1.

### Surgery

Surgery was undertaken using a surgeon-specific longitudinal or oblique incision over the injured clavicle. When possible, inter-fragmentary lag screw was used for temporary fixation followed by rigid fixation with a reconstruction plate, locking reconstruction plate (Synthes, Bochum, Germany), dynamic compression plate (DCP), or pre-contoured locking compression plate (LCP superior clavicle plate, Synthes, Bochum, Germany). The plates were selected according to the surgeon preference. The plates were contoured by the surgeon to fit the shape of the clavicle and were all positioned on the superior surface of the bone with a minimum of three bicortical screws in both the proximal and distal end.

### Postoperative treatment and follow-up

Postoperative treatment was active non-weight-bearing motion exercises of the shoulder within the first 8 weeks. Patients were allowed to start weight-bearing motion exercises after satisfactory radiographic findings after 8 weeks follow-up.

All the operated clavicles were evaluated radiographically in the anteroposterior (AP) view and 30° caudal and cephalic tilt (CC) view immediately and every month after operation until union was achieved. Bone union is defined as cortical bridging of at least three of cortices radiographically and no tenderness at the fracture site clinically [13].

### Treatment outcome assessments

**Fig. 2:**
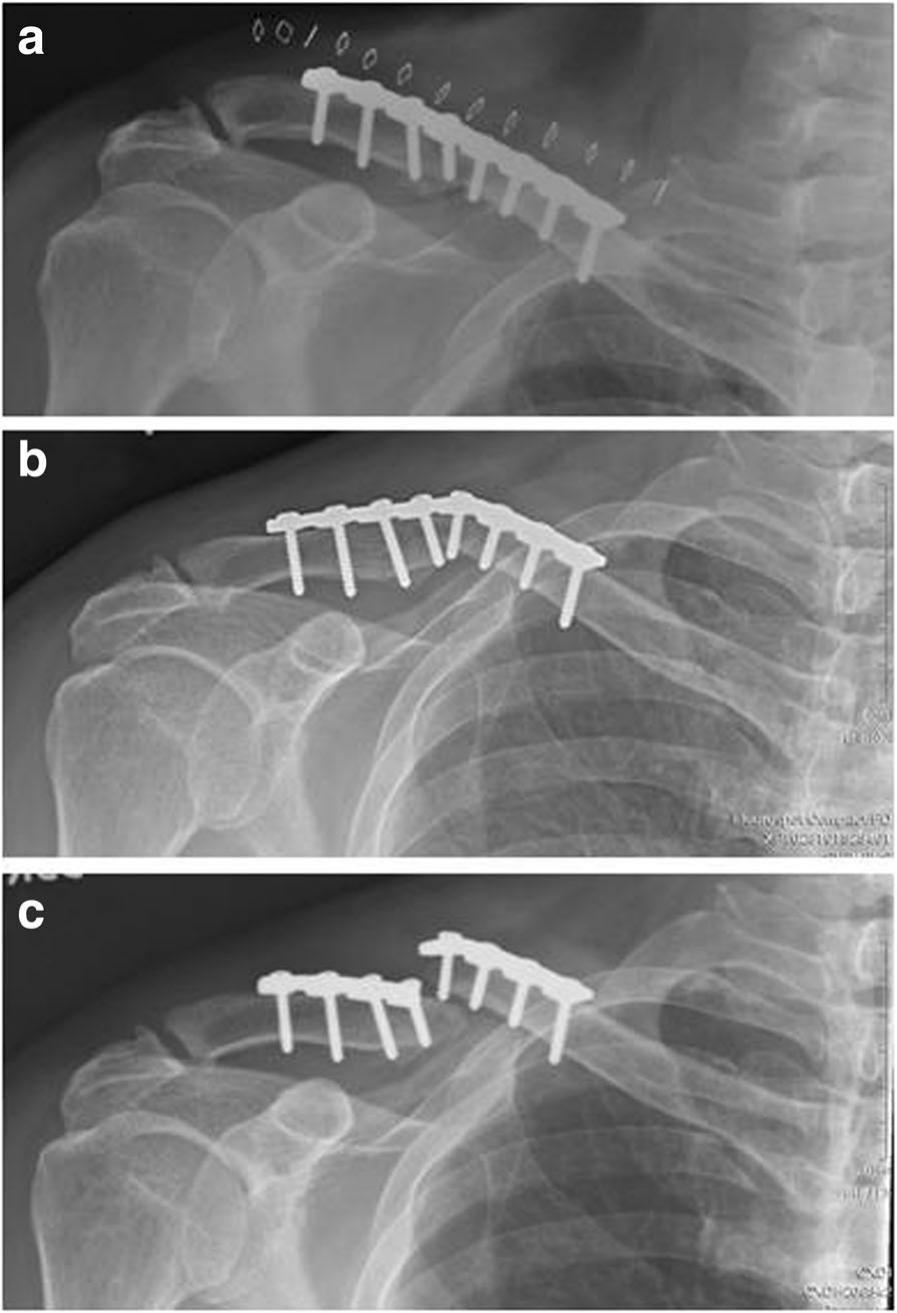
**a** 56- year-old male patient with midshaft clavilcular fracture after open reduction and fixation with reconstruction plate. **b** Obvious bending of plate was observed at the 6-week follow-up radiograph. **c** Complete break of plate was found at the 8-week follow-up radiograph. The patient received reoperation and fixation with locking plate. Uneventful bone union was found 12 weeks later

Implant failure was defined as the occurrence of any of the following events: (1) dislodge or loosening of the screws, (2) breakage of the plate, and (3) any angle change of the plate compared to postoperative radiographies during the 3-month postoperative follow-up [8] (Fig. 2). Nonunion was defined as no progression of radiographic healing in the first 12 months of treatment. Reoperation was defined as the requirement of a second operation for implant failure or nonunion. Patients who requested elective removal of an implant were not included in the analysis.

### Statistical analysis

Data analysis was performed using SPSS software (Version 19.0; Chicago, Illinois). Univariate analysis was performed using frequencies for descriptive statistics. Chi-square and Fisher’s exact test were used in the analysis of categorical variables. Logistic regression was performed to evaluate the risk predictors of implant failure. The covariates with a *P* value ≤ 0.1 in the univariate analysis were included in the multivariate analysis. Correlations were considered significant if *P* values were less than 0.05 (two-sided).

## Results

Three hundred nineteen consecutive patients received operation for acute clavicle fracture during the period Jan 2012 to July 2017 in our hospital, of whom 263 patients had a midshaft clavicular fracture that was treated with open reduction and plating. Thirty-seven patients were excluded during follow-up because of fracture nonunion (*n* = 4) and a follow-up time shorter than 12 weeks (*n* = 33). The remaining 226 patients with 226 midshaft clavicular fractures were included in the final analysis (Fig. 1). There were 148 males (66%) and 78 females (35%) with a mean age at operation of 43.5 years (13 to 87). Their mean follow-up time was 60.3 weeks (27 to 274). One hundred fifty-nine patients were treated with reconstruction plates and 67 patients were treated with non-reconstruction plates. Among the 67 patients, 19 were treated with locking reconstruction plates, 20 with DCPs, and 28 with pre-contoured LCPs.

Thirty-one implant failure cases were found among the 226 patients in the 3-month postoperative period, and thus the implant failure rate was 13.7%. The implant failure rates of reconstruction plates and non-reconstruction plates were 16.9% and 6.0% respectively. A detailed list of implant failure causes are provided in Table 1. Eight (2.6%) of the patients received revision surgeries with non-reconstruction plate fixation. All of them achieved uneventful bone union after at least 22 weeks follow-up. For the other 23 patients with implant failure who did not receive revision surgery, one patient did not achieve bone union until the last follow-up OPD at 6 months after operation. Twenty-two patients experienced delayed union and various degrees of mal-union. The average union time was 35 weeks. All of 195 patients without implant failure achieved uneventful bone union with average union time of 12 weeks. The predictors of implant failure risk based on demographic data and implant failure rate are summarized in Table 2. Significantly higher failure rates were found in the 15-2C fracture type, reconstruction plate, and male gender groups.

**Table 1:** Details of implant failure

Type of treatment failure	Total subjects *N* = 226 (%)
Implant failure	31 (13.7)
Plate breakage	4 (1.8)
Bending of plate	18 (8.0)
Screw loosening	9 (3.9)

**Table 2:** Basic characteristic of 226 mid-shaft clavicular fractures treated with open reduction and plate fixation

Variables	Total (%) *N* = 226	Union (%) *N* = 195	Implant failure (%) *N* = 31	*P* value
Age, years				0.318
< 60	184 (81)	161 (83)	23(74)	
≥ 60	42 (19)	34 (17)	8 (26)	
Gender				*0*.*024*
Male	148 (66)	122 (63)	26 (84)	
Female	78 (35)	73 (37)	5 (16)	
Injured side				0.327
Right	90 (40)	75 (39)	15 (48)	
Left	136 (60)	120 (62)	16 (52)	
AO Classification				< *0*.*001*
15-2A	106 (47)	101 (52)	5 (16)	
15-2B	61 (27)	52 (27)	9 (29)	
15-2C	59 (26)	42 (22)	17 (55)	
Implant				
Non-recon	67 (30)	63 (32)	4 (13)	*0*.*033*
Recon	159 (70)	132 (68)	27 (87)	

In the regression analysis of significant risk factors, gender, AO/OTA fracture classification, and implant type met the criteria and were included in the multivariate analysis. AO/OTA 15-2C fracture classification and the use of reconstruction plates were found to have a statistically significant effect on implant failure rates after multivariate adjustment (Table 3).

**Table 3:** Multivariant regression of predictors for implant failure

Risk factors	Crude OR (95% C.I.)	*P* value	Adjusted OR (95% C.I.)	*P* value
Age (years)				
< 60	1.00 (Ref.)			
≥ 60	1.467 (0.679–3.993)	0.269		
Gender				
Female	1.00 (Ref.)		1.00 (Ref.)	
Male	3.111 (1.145–8.459)	*0*.*026*	2.249 (0.770–6.569)	0.138
Injured side				
Right	1.00 (Ref.)			
Left	0.667 (0.311–1.427)	0.296		
AO/OTA classification				
15-2A	1.00 (Ref.)		1.00 (Ref.)	
15-2B	3.496 (1.115–10.967)	*0*.*032*	2.677 (0.833–8.605)	0.088
15-2C	8.176 (2.832–23.601)	< *0*.*001*	11.401 (3.765–35.366)	< *0*.*001*
Plate type				
Non-recon	1.00 (Ref.)		1.00 (Ref.)	
Recon	3.222 (1.081–9.602)	*0*.*036*	6.006 (1.788–20.173)	*0*.*004*

Stratified analysis was performed to determine the relationship between implant failure rates for reconstruction plate and pre-contoured LCP in different AO/OTA classifications. Reconstruction plates had a significantly higher failure rate than LCPs for comminuted midshaft clavicular fracture (AO/OTA 15-2C) (*P* < 0.001). However, the difference was not significant for AO/OTA 15-2A and 2B fracture classifications (Table 4).

**Table 4:** The relationship between implant failure rates for reconstruction plate and pre-contoured LCP in different AO/OTA classifications

	Union *N* = 159	Implant failure *N* = 31	*P* value
AO/OTA 15-2A			0.613
Non-recon (%)	27 (93)	2 (7)	
Recon (%)	74 (96)	3 (4)	
AO/OTA 15-2B			1.000
Non-recon (%)	8 (89)	1 (11)	
Recon (%)	44 (85)	8 (15)	
AO/OTA 15-2C			
Non-recon (%)	28 (97)	1 (3)	< *0*.*001*
Recon (%)	14 (47)	16 (53)	

## Discussion

The aim of this investigation was to determine the main risk factors of implant failure for midshaft clavicular fractures and to compare the implant failure rates between the reconstruction plate and non-reconstruction plate based on different fracture classifications. Our finding showed that (1) significant risk factors for implant failure or midshaft clavicular fractures were AO/OTA classification and plate type, and (2) reconstruction plates had a significantly higher failure rate than non-reconstruction plates in comminuted (AO/OTA 15-2C) fractures.

Reconstruction plates are widely used for mid-clavicular fracture fixation, but their implant failure rates vary considerably in the literature. A review of previous studies is summarized in Table 5. Studies with smaller case numbers reported lower implant failure rates than in our study [8, 9, 14–16]. However, two studies with over 100 cases reported implant failure rates of 12% and 12.6%, which were similar to our results [5, 7].

**Table 5:** Implant failure rates for reconstruction plates in the literature

Author, year	Patient number	Plate location	AO/OTA classification	Implant failure rate
Galdi et al. 2013 [12]	18	Anterior	B1, B2, and B3	0%
Mirzatolooei et al. 2011 [9]	26	Superior	Not mentioned	7%
Tarng et al. 2012 [14]	32	Superior	B1, B2, and B3	Not mentioned
Kulshrestha et al. 2011 [13]	45	Superior	B1, B2, and B3	Not mentioned
Gilde et al. 2014 [8]	71	Anterior	B1, B2, and B3	8.5%
Chen et al. 2012 [10]	84	Not mentioned	B1 and B2	7%
Woltz et al. 2016 [6]	111	Superior and anterior	B1, B2, and B3	12.6%
Shin et al. 2012 [7]	125	Superior	B2 and B3	12%

Locking reconstruction plates is thought to achieve a better outcome owing to their increased angular stability for screws and plate. In the literature, locking reconstruction plates were reported to have implant failure rates of 7% and 8.5% in studies by Cho and Liu, respectively [17, 18]. Cho and his colleagues compared the complication rates between reconstruction plate and locking reconstruction plate for the treatment of midshaft clavicular fracture and found no significant difference in implant failure rates between the two types of implants [17]. Liu and his colleagues compared the operative outcomes between intramedullary nails and locking reconstruction plates in 51 and 59 midshaft clavicular fractures and found no significant differences in radiographic and functional outcomes. DCPs were suggested to have a lower implant failure rate compared with reconstruction plates owing to their superior strength. However, a wide range of implant failure rates for DCP have been reported in the literature [19]. Ashman et al. compared operative outcomes of 143 midshaft clavicular fractures treated with 51 DCPs and 92 reconstruction plates and reported that the implant failure rate of DCP was 1.4%, which was significantly lower than that of reconstruction plates [19]. Lai et al. compared operative outcomes of 18 DCPs and 22 LCPs and found no differences in operative time, blood loss, complication rate, hospital stay, and union rate between these two implants. DCP was reported to have an implant failure rate of 16.7%, which was much higher than that found in a study by Ashman [20].

Pre-contoured LCPs were reported to have good outcomes, with implant failure rates as low as 1.1% and 0.6% in two multicenter randomized controlled studies [2, 3]. The two aforementioned studies compared the outcomes between non-operative treatment and fixation of pre-contoured LCP and found that patients treated with pre-contoured LCP had significantly better functional and radiographic outcomes than patients who received non-operative treatment. Our study demonstrated that all these non-reconstruction plates had a significantly lower implant failure rate than that of reconstruction plates, especially in comminuted (AO/OTA 15-2C) fractures.

Biomechanical studies also showed that DCPs and pre-contoured LCPs had higher compression stiffness, torsion stiffness, and cantilever bending failure load than reconstruction plates [21, 22], while locking reconstruction plates only had higher compression stiffness than reconstruction plates [23]. This may explain why non-reconstruction plate had lower implant failure rate in the present study.

In AO/OTA 15-2A and 15-2B fractures, there was no significant difference in implant failure rates between reconstruction plates and non-reconstruction plate. This finding suggests that the strength of reconstruction plates may be sufficient for non-comminuted fractures, which can be relatively easily fixed by anatomic reduction. In comminuted (AO/OTA15-2C) fractures, the strength of the plate is the most important factor for fracture stability, especially when anatomic reduction is hard to achieve.

There were several limitations in this study. First, the operations were not performed by a single surgeon. The operative skills of surgeons may have been varied and this could have affected the treatment outcome. Second, the bending and breaking of plates were only evaluated by radiography, so some implant failures could have been missed. However, four implants failed in our study, which was a higher rate than those reported in the literature. The higher implant failure rate in our study may be due to our inclusion of plate bending as a cause of implant failure, whereas most previous studies did not include this type [7, 8, 15, 16]. Third, due to the retrospective nature of this study, factors that may confound the effects of treatment method on outcomes, such as bone density and bone quality, BMI, mechanism of injury, smoking, dominant arm, mental status, shoulder function, and plate irritation, were not included in our statistical analysis. Fourth, the numbers of cases in the locking reconstruction plate and DCP groups were too small to achieve statistical significance. Further studies with a larger population should be done to confirm these results.

## Conclusion

We found that patients with comminuted midshaft clavicular (AO/OTA 15-2C) fractures treated with reconstruction plates had very high implant failure rates compared to non-reconstruction plates. These findings may provide useful information that orthopedic doctors can use to identify patients who have a higher risk for implant failure. We also suggested that patients with comminuted midshaft clavicular (AO/OTA 15-2C) fractures treated with reconstruction plates need more protection and more frequent follow-up in the postoperative period.

## Data Availability

The datasets used and analyzed during the current study are available from the corresponding author on reasonable request.

## Abbreviations

AO/OTA: Arbeitsgemeinschaft für Osteosynthesefragen/Orthopedic Trauma Association
AP: Anteroposterior
CC: Caudal and cephalic tilt
DCP: Dynamic compression plate
LCP: Locking compression plate
ORIF: Open reduction and internal fixation
